# Active Immune Phenotype in Head and Neck Cancer: Reevaluating the Iso-Effect Fractionation Based on the Linear Quadratic (LQ) Model—A Narrative Review

**DOI:** 10.3390/curroncol30050362

**Published:** 2023-05-08

**Authors:** Camil Ciprian Mireștean, Roxana Irina Iancu, Dragoș Petru Teodor Iancu

**Affiliations:** 1 Department of Oncology and Radiotherapy, University of Medicine and Pharmacy Craiova, 200349 Craiova, Romania; 2 Railways Clinical Hospital Iasi, Department of Surgery, 700506 Iași, Romania; 3 Faculty of Dental Medicine, Oral Pathology Department, “Gr. T. Popa” University of Medicine and Pharmacy, 700115 Iași, Romania; 4 Department of Clinical Laboratory, “St. Spiridon” Emergency Universitary Hospital, 700111 Iași, Romania; 5 Faculty of Medicine, Oncology and Radiotherapy Department, “Gr. T. Popa” University of Medicine and Pharmacy, 700115 Iași, Romania; 6 Regional Institute of Oncology, Department of Radiation Oncology, 700483 Iași, Romania

**Keywords:** HNSCC, HPV, head and neck cancer, radio-sensitivity, radio-resistance, immune active, immune exhausted, linear quadratic (LQ) model

## Abstract

Altered fractionation concepts and especially moderate hypo-fractionation are evaluated as alternatives to standard treatment for head and neck squamous cell carcinoma (HNSCC), associated with or not concurrent with or sequential to chemotherapy. The calculation of the iso-equivalent dose regimens has as its starting point the linear quadratic (LQ) formalism traditionally based on the “4Rs” of radiobiology. The higher rates of therapeutic failure after radiotherapy of HNSCC are associated with the heterogeneity of radio-sensibility. The identification of genetic signatures and radio-resistance scores aims to improve the therapeutic ratio of radiotherapy and to conceptualize personalized fractionation schemes. The new data regarding the involvement of the sixth “R” of radiobiology in HNSCC, especially for the HPV-driven subtype, but also for the “immune active” minority of HPV-negative HNSCCs, bring to the fore a multifactorial variation of the *α/β* ratio. The involvement of the antitumor immune response and the dose/fractionation/volume factors as well as the therapeutic sequence in the case of new multimodal treatments including immune checkpoint inhibitors (ICIs) could be included as an additional term in the quadratic linear formalism especially for hypo-fractionation regimens. This term should take into account the dual immunomodulatory effect (immunosuppressant and stimulator of antitumor immunity) of radiotherapy, which varies from case to case and can bring benefit or a detrimental effect.

## 1. Introduction

Head and neck cancers are generally aggressive tumors that are resistant to non-surgical multimodal treatment. The concepts of altered fractionation (hyper-fractionation, accelerated fractionation, hypo-fractionation) for curative intent in association with pharmacological agents—among which we mention the anti-alkylant cisplatin; cetuximab, a molecular therapy target anti-epidermal growth factor receptor (EGFR) monoclonal antibody; or nimorazole, a radio-sensitizer nitrosamine—aim to activate a synergistic effect and increase the response rates to multimodal treatment. The identification of a subtype of squamous cell carcinoma of the head and neck (HNSCC) associated with the human papillomavirus (HPV) with a different treatment response pattern and also the advances in radiobiology that extend beyond the traditional “4R” (repair, redistribution, re-oxygenation, repopulation) concept—the newer “2Rs” (intrinsic radio-sensitivity and reactivation of the antitumor immune response) in the current “6R” concept—bring new challenges in the application of the linear quadratic (LQ) model for the calculation of iso-equivalent fractionation schemes. We propose to summarize the concepts that will be the basis for the reconsideration of the LQ formalism for the category of “active immune class” of HNSCC regardless of HPV status [1,2,3] (Equation (1)).

(1) $$BED=nd\left[1+\frac{d}{\frac{\alpha }{\beta }}\right]$$

*BED*—biological effective dose;
*n*—number of radiotherapy fractions;
*d*—dose per fraction;
*α*/*β*—a ratio correlated with the cellular response to the size of the irradiation fractions, being dependent on the linear and quadratic component of the cellular response.

The quadratic linear formalism proposes a relationship between cell survival and delivered dose in radiotherapy, offering an ideal model for equating the biological effect of the fractionation regimes. However, the variation in the intrinsic and extrinsic radio-sensitivity of tumors as well as of healthy tissues makes this model an ideal and imperfect model. Potential challenges including the use of this model at very low and very high dose per fraction and possible confounding factors must be evaluated before applying this model in clinical routines. A low *α*/*β* ratio value, usually between 1 and 5, is associated with late-responding tissue. Squamous cell carcinoma is associated with high values of the *α*/*β* ratio and is correlated with acute responses to irradiation and reduced sensitivity to the fractionation regime. Lower values of *α*/*β* are associated with a predominance of the quadratic effect, in which case the tissues are spared at doses per fraction below 2Gy. High values of *α*/*β* (6–14 Gy) mean a predominance of the linear component. A rough approximation proposes the value *α*/*β* = 10 for the tumor and acute effects and *α*/*β* = 3 for healthy tissues and late effects [4,5,6,7].

In HNSCC, a dose of 70 Gy in 33–35 fractions delivered on gross disease (primary tumor and involved lymph nodes) is considered necessary in definitive settings, and 66Gy in 33 fractions is usually preferred as adjuvant treatment. Noninvolved lymph node irradiation is also administered in dose regimens of 45 to 60 Gy in a standard fractionation regimen, with 50Gy and 60Gy dose levels being the most frequently proposed for low risk and high risk of tumor involvement, respectively [8,9]. Currently, regardless of HPV status, concurrent radio-chemotherapy with cisplatin or cetuximab (an anti-epidermal growth factor receptor (EGF) monoclonal antibody, in cases not eligible for cisplatin) is considered the standard treatment. Induction chemotherapy followed by concurrent chemo-radiotherapy is also an accepted strategy but is still controversial as to its benefits. Radiobiological mechanisms that lead to the loss of the biological effect may result in treatment prolongation. A short treatment (4–8 days) prolongation and a longer treatment delay (>8 days) are associated with a reduction in survival at 4 years of 4 and 12%, respectively. However, extending the treatment for <4 days does not seem to have a detrimental effect [10,11,12].

## 2. Materials and Methods

We performed a search in the PubMed/MEDLINE database using the keywords radiotherapy, radio-chemotherapy, HPV, head and neck, HNSCC, hypo-fractionated, quadratic linear model, immunotherapy, and immune checkpoint inhibitors.

## 3. HPV-Driven HNSCC—Implication in Radio-Sensitivity

### 3.1. The Molecular and Immune Pattern of HPV-Driven HNSCC

The higher rates of therapeutic failure and the limited number of responses to immune checkpoint inhibitor (ICI) therapy justify the design of a genetic prognostic index in order to predict the response to immunotherapy in HNSCC. The purpose of the immune-related gene prognostic index (IRGPI) is to identify the immune and molecular characteristics of HNSCC that will influence ICI therapy response rates. In all, 22 genes related to antitumor immunity were evaluated, and 3 genes (SFRP4, CPXM1, and COL5A1) were chosen for the construction of the IRGPI. The IRGPI value was inversely correlated with overall survival (OS). Reduced values of the IRGPI have been associated with DNA damage repair mechanisms. Higher levels of CD8+ and CD4+ T lymphocytes and M1 macrophages and lower expression of TP53 seem to be associated with a favorable response to ICIs. The aggressive phenotype is associated with higher rates of TP53 and PIK3CA mutations and also with the infiltration of B cells and M0 and M2 macrophages [13].

The subtype of head and neck cancers associated with human papillomavirus (HPV) infection is currently considered an independent subtype of HNSCC, but more recently, HPV-positive head and neck cancers could be divided into HPV-DNA-positive tumors and HPV-expressing RNA and proteins. Even though the two subtypes are reported to be different, there are many similarities between HPV-DNA-positive HNSCC and HNSCC associated with smoking. With an increasing incidence, the so-called “HPV-driven” subtypes represent up to 25% of HNSCCs, but oropharynx squamous cell carcinomas (OPCCs)—the oropharynx is the most frequent location of this subtype—benefit from a new staging in the eighth-edition TNM classification. The incidence of the HPV-driven subtype varies from 20% to 60%, with the countries of Western Europe and North America having increased incidences. Both the prognosis and the response to treatment seem to be superior in this subcategory of HNSCC. Among the particularities of HPV-driven HNSCC, especially for OPSCCs, there is a frequent involvement of the lymph nodes in young, nonsmoking patients, but certain socio-sexual conditions make the appearance of this subtype at older ages possible. The increased sensitivity to irradiation and the favorable prognosis justified the initiation of de-escalation trials. Considering the differences in TNM staging compared to the subtype related to a long history of smoking and chronic alcohol consumption, identification tests for the HPV-driven subtype should be performed in all cases of OPSCC according to the recommendations of the College of American Pathologists [14,15,16]. Four randomized trials (RTOG9003, DAHANCA 6&7, RTOG0129, ARTSCAN) were included in the Meta-Analysis of Radiotherapy in Carcinomas of Head and neck (MARCH) that evaluated the prognostic impact of HPV-associated p16-expression, smoking, and an altered fractionation radiotherapy regimen. The study group included 465 patients with p16-positive tumors and 350 p16-negative cases, representing 57% and 43%, respectively. A 32.1% benefit in OS at 10 years was associated with p16-positive tumors. The negative effect of smoking in the p16-negative group should also be mentioned, but there was a lack of impact for altered irradiation fractionation regimens [17].

### 3.2. Radiosensitivity in HPV-Driven HNSCC—From the Classical LQ Model to Modern Scores and Indexes

Torres-Roca et al. proposed a radiation sensitivity index (RSI), in order to modulate treatment for a subgroup of 40–45% of rectal cancer cases considered not respondent to neo-adjuvant chemo-radiotherapy. The authors also noted a 20% complete pathological response, considering the critical role of radio-sensitivity in the tumor control of locally advanced rectal adenocarcinoma [18]. Extending the applicability of the RSI to head and neck cancers, the authors propose the association of RSI with known prognostic factors including HPV status in order to identify cases that would benefit from an intensification of treatment or radio-sensitizing agents. RSI is based on a ten-gene network, trained and perfected on 48 HNSCC cell lines and subsequently validated on 5 patient lots including 621 cases. Even if it is not yet considered translatable into clinical practice, RSI opens new horizons in molecular-guided radiotherapy based on radio-sensitivity criteria [15,16,17,18].

Starting from this concept of different radio-sensitivities, Foy et al. aimed to evaluate the molecular factors associated with these differences in radio-sensitivity in HPV-HNSCC patients. The authors proposed a RadR radio-resistance score based on a training set and a validation set that included HPV-negative HNSCCs from The Cancer Genome Atlas HNSCC cell lines with known radio-sensibility levels [13,19,20]. Patterns of protein expression, drug sensitivity, and biological hallmarks were also evaluated in an integrated analysis together with the RadR score, in addition to genetic alteration being correlated with CCND1 amplification, fibronectin expression, epithelial–mesenchymal transition, and sensitivity to an HSP90 inhibitor. The association of CCND1 with Von Hippel–Lindau syndrome, multiple myeloma, and malignant melanoma with unfavorable response to immunotherapy characterized by an immunosuppressive microenvironment is worth mentioning. The RadR score is also heterogeneous between the HPV-negative HNSCC subtypes, but for HPV-driven HNSCC, the RadR score is lower, which corresponds to an increased level of radio-sensitivity. The genomic-adjusted radiation dose (GARD) concept proposes guiding the radiation dose according to the radio-sensitivity levels evaluated on 20 disease sites treated with standard radiotherapy. Cervical cancer and OPSCC were associated with the highest GARD score and the highest radio-sensitivity levels [21,22,23].

### 3.3. The Genetic and Molecular Profile Orchestrates the Radiosensitivity of HPV-Driven HNSCC

The effects of HPV on the repair of radiation-induced cellular damage are controversial. In the case of double-strand breaks (DSBs) there is a deficit in repairing the lesions; however, for single-strand break (SSB) lesions, there is a possible repair mechanism involving the E6 oncoprotein. XRCC1, DNA polymerase β, PNKP and PARP-1, all factors involved in SSB repair, appear to be upregulated in HPV-driven HNSCC. A defect in homologous recombination (HR) that also affects the expression of some factors involved in DSB repair promotes radio-sensitivity. However, the lack of a favorable effect of poly (ADP-ribose) polymerase (PARP) inhibition on HPV-driven HNSCC suggests the minimal involvement of the HR mechanism in radio-sensitivity. Non-homologous end joining (NHEJ), HR, mismatch repair, and the involvement of the E6 and E7 oncoproteins all have an overwhelming role in the high radio-sensitivity of HPV+ HNSCC. In addition, the radio-sensitizing role of the ataxia telangiectasia gene (ATM) is considered minimal in the case of HPV-driven HNSCC compared to the subtype of HNSCC related to smoking. Köcher et al. mentions that a lack of effectiveness in the ATM-orchestrated DNA damage response contributes to the DNA repair defect in HPV + HNSCC. TP53 mutation is frequently associated with radio resistance, but HPV-driven HNSCCs are associated with wild-type TP53. Consequently, it is unlikely that in this case radio-sensitivity is dependent on this pathway, so strategies to increase radio-sensitivity by using the inhibition of proteasomal degradation cannot be applied to HPV+ HNSCCs. The hypoxia-related proteins CAIX or HIF-1α are considered biomarkers of radio resistance in HPV+ HNSCC. CAIX or microvascular density does not seem to be correlated with the HPV status of HNSCC, and in the case of HIF-1α, the correlation with HPV+ OPSCC and involvement in radio-sensitivity has not yet been elucidated [24,25,26,27,28,29].

### 3.4. Tumor Metabolism and Hypoxia—Other Players in the Radiosensitivity of HPV-Driven HNSCC

Analyzing the tumor metabolism, there are major differences between smoking-related HNSCC tumor cells and HPV-driven HNSCC cells. In the case of HPV-HNSCC the Warburg effect, an increased glucose uptake associated with the fermentation of glucose to lactate, is the predominant metabolic pathway; however, HPV+ cancers produce energy using mitochondrial oxidative phosphorylation. The strategy of radio sensitization using the biguanide metformin, a glycolytic inhibitor, or the association of both agents thus proves useless in the radio sensitization of the HPV+ subtype of HNSCC [30,31]. Similar patterns of hypoxia-induced gene upregulation are mentioned by Sørensen et al. in both subtypes of HNSCC. However, the DAHANCA 5 randomized trial does not highlight a benefit of nimorazole treatment for HPV-driven HNSCC. The study on cell lines proposed by the authors identified a similar radio sensitizing effect in both subtypes of HNSCC. The radio sensitizing effect of nimorazole exists, but it is probably considered minimal taking into account the higher radio-sensitivity of HPV-driven HNSCC. The interactions among the glycolysis process, the signaling pathways, and the tumor microenvironment are also associated with chemotherapy resistance. The different metabolic pathway that characterizes HNSCC could also justify the variation in chemo-sensitivity [32,33,34].

### 3.5. Chemotherapy and Target Therapy in HPV-Driven HNSCC—New Radiobiological Implications

Analyzing six HNSCC cell lines of which three were HPV negative and three were HPV positive, Reid et al. evaluated the radio-sensitivity after repeated irradiation simulating fractionated radiotherapy. Even if the HPV-positive cell lines demonstrated a superior radio-sensitivity, repeated fractionations resulted in radio-sensitivity changes in both subtypes of cell lines. This variation in radio-sensitivity during treatment justifies the difficulties in implementing de-escalation strategies. The radiobiological heterogeneity of HPV-driven HNSCCs regarding *α*/*β* ratios values and the radio-sensitivity variation during radiotherapy treatment are arguments against the de-escalation of radiation treatment based only on HPV status. The estimation of *α*/*β* ratios highlights variations from 1.01 to 40.68 Gy: there are cell lines that act as late-responding tissues at *α*/*β* ratios < 6 but also cell lines that behave as acute-responding tissues at *α*/*β* ratios between 8.19 and 34.1 Gy. Heterogeneity in radio-sensitivity was confirmed by van Leeuwen and colleagues, who estimated in a systematic review *α*/*β* ratios varying from 8.36 Gy to 30.0 Gy. In this context, the individual determination of radio-sensitivity could be essential for improving treatment performance. In the case of HPV-positive cell lines, a resistance to cisplatin is highlighted, and the response to cetuximab and radiotherapy does not seem to differ compared to the HPV subtype according to the reports of Nagel et al. [23,24,25]. Enhanced apoptosis rates and the decreased expression of E6 and E7 oncoproteins were considered the arguments for the increased sensitivity of HPV-driven HNSCC to cisplatin. Chemo-radiotherapy in HNSCC can be associated with lipopenia, which can still be associated with immunosuppression and a bad prognosis. For this reason, the general concept of potentiation of the antitumor immune response is subject to relativity related to factors such as the dose and treatment regimen. Metronomic chemotherapy also reaches new horizons in this context of immunomodulation. Even if induction chemotherapy is a controversial subject in HNSCC, for this tumor subtype, this regimen seems to acquire new values, both for reducing the risk of metastases in de-escalation trials and as a potential biomarker to guide subsequent treatment [34,35,36,37]. The high rates of G2/M arrest, the shortage of stem cells, and a less hypoxic microenvironment are mechanisms involved in determining the radio-sensitivity of HPV-driven HNSCC. The DNA lesions produced by radio-chemotherapy generate the release of neo-antigens and tumor antigens and generate the effect of “in situ vaccination” [38,39].

## 4. Immune Response—Key of Future Radiobiological Models in Head and Neck Cancers: From Mechanisms to Clinical Practice

Traditionally, it is considered that anticancer treatments act through the effect of DNA damage in three phases: physical, chemical, and cellular. Fractional irradiation is supported by the classic concept including the “4Rs” of radiobiology and the counteracting the mechanisms of radio-resistance—the repair of radiation-induced injuries; the recovery of cells for their arrest in radiosensitive phases including mitosis and the increase in the chance of arresting the cells coming out of the S phase, considered radio-resistance; cellular repopulation between fractions; and re-oxygenation—with the aim of increasing radio-sensitivity. The concept of intrinsic radio-sensitivity introduces a new, decisive factor of treatment response. The high rates of G2/M arrest, the shortage of stem cells, and a less hypoxic microenvironment are mechanisms involved in determining the radio-sensitivity of HPV-driven HNSCC. The DNA lesions produced by radio-chemotherapy generate the release of neo-antigens and tumor antigens and generate the effect of “in situ vaccination” [26,38,39].

The sixth “R”, the reactivation of the immune response becomes all the more important since immunotherapy with immune checkpoint inhibitors has demonstrated a synergistic potential with radiotherapy. The effect of “in situ vaccination” and the systemic effect induced by “abscopal” radiotherapy are determined by the immune modulation induced by irradiation. Fractionation, dose, and timing are three essential factors that define the immunosuppression/immunopotentiation balance induced by irradiation. The modulation of the tumor microenvironment (TME) by irradiation is also considered a cause of the radio-sensitivity variation. The TME modulation by irradiation includes the stimulation of some immune mechanisms that act directly or indirectly at the tumor level by releasing cytokines, chemokines, and enzymes. The different radio-resistances of some components of the TME and their different effects (immunosuppressor or stimulator of antitumor immunity) are the basis of the opposite effects of irradiation at the level of the TME. Among the factors associated with immunosuppression, we mention MDSCs, Tregs, and natural killer cells (NKs), and CD8+ T lymphocytes are stimulators of immune-mediated tumor evasion. NKs are considered among the most radiosensitive immune cells, and cancer-associated fibroblasts (CAFs) and dendritic cells (DCs) are considered much more radio-resistant. Furthermore, Tregs are considered more radio-resistant than other types of T lymphocytes including the CD8+ subtype [40,41,42,43,44]. Understanding these differences in radio-sensitivity in order to translate them into clinical practice also justifies the concept of “hybrid” radiotherapy, including a combination of low-dose and high-dose radiotherapy [1,45].

Targeting the cyclic GMP–AMP synthase/stimulator of interferon genes (cGAS/STING) pathway is also considered a future strategy in immune-modulated radio-sensitization [46]. A finding by Spanos et al., who evaluated the in vitro response of both HPV+ and HPV-HNSCCs in immune-competent mice and immunosuppressed mice, indicated a more favorable response to irradiation in the case of HPV+ tumors only in the case of immune-competent mice but not in mice lacking adaptive immunity. HPV-specific immune mechanisms are involved in tumor clearance; the in vivo antigen-specific antitumor response to HPV+ is mediated by CD4+ and CD8+ T lymphocytes. The immune response against HPV+ HNSCC involves the E7 oncoprotein, which is considered a possible target for E7-specific immunotherapy. The identification of mechanisms of antigen-processing upregulation related to HPV infection is considered another possible strategy in order to improve the immune response against HPV-driven HNSCC [47,48,49].

The increase in antigen presentation and recognition by the host’s immune system, phenomena linked by cell death related to irradiation, seem to have a decisive role in the induction of an antitumor immune response. The inflammation associated with irradiation and the release of cytokines IL-6 and IL-8 and tumor necrosis factor (TNF)-α are involved in the activation of the immune system. Damage-associated molecular patterns (DAMPs) are endogenous molecules released by dying cells that initiate a reaction of the host immune system. Their release is activated by radiotherapy, chemotherapy, and also by oncolytic viruses. An association between the viral neoantigen and DAMPs is considered essential to generate an immune response that defeats the tumor’s immune-escape mechanisms [50,51,52].

CD39 and LAG3, PD1, TIGIT, and TIM3, considered T-cell exhaustion markers, are identified in a large proportion of HPV+ HNSCC cases but not in the HPV− subtype. The presence of T-cell exhaustion markers is considered a biomarker of a T-cell-inflamed tumor phenotype correlated with a favorable prognosis. The difference between two subtypes of HPV+ OPSCCs classified according to tumor-infiltrating lymphocytes (TILs) demonstrates the biomarker value of this parameter of cellular immunity in HPV+ HNSCC as well. Overall survival (OS) rates at 3 years of 94.72% and 56%, respectively, for TILS^high^ vs. TILS^low^ HPV+ cancers and similar OS rates at 3 years (51%) for HPV− HNSCC clearly demonstrate the association of the immune-mediated response with the subtype HPV+. Infiltration of the stroma with CD8+ T lymphocytes is considered by Oguejiofor et al. as a positive prognostic factor in HPV-driven HNSCC. CD4, but not FoxP3 T regulatory cells, was identified in HNSCC HPV+ stromal infiltrates. A CD8+ T cell cutoff level > 24% is identified by Wansom and collaborators as a favorable prognostic factor. CD3+ and CD8+ lymphocytes infiltrated in the tumor border core included in an immune score are considered more significant biomarkers for treatment de-escalation strategies than p16 status. Disproportional enrichment of FoxP3+ CD4+ regulatory T is mentioned by Park et al. as a decisive factor in the creation of an immune-suppressive TME in HNSCC. The authors mentioned the involvement of this Treg in the possible unfavorable response to immunotherapy. However, the HPV-driven HNSCC cases are notable for a high proportion of both Treg and CD8+. Surprisingly, although they are considered immunosuppressive, increased levels of FoxP3+ T cells were correlated with better disease-free survival (DSF) and OS. The highest median Treg/CD8+ T cell ratio and high levels of CD8+ were identified by Mandal et al. in HNSCC, regardless of HPV status. A higher level of NK cell infiltration in HNSCC is a positive prognostic factor, but the genetic signature of smoking is associated with a poor immune TME and a worse prognosis [53,54,55,56,57].

The increased response rate to pembrolizumab treatment in cases of HPV+ HNSCC programmed cell death Protein 1 (PD-1)–positive patients, 25% vs. only 14% for HPV-HNSCC cases, also suggests the involvement of HPV status in the response to a possible association of PD-1 inhibitors and radiotherapy. Higher levels of myeloid-derived suppressor cells (MDSCs) are considered factors associated with immunosuppression in HNSCC. Regardless of HPV status, higher levels of MDSCs are associated with advanced stages (III–IV). Jayaraman et al. mentioned the potential of transforming a tumor suppressor in association with TGF-β1 (TGFβ-MDSC) in a stimulation of the immune-mediated rejection of the tumor. The upregulation of FAS potentiated by radiotherapy in combination with intratumoral injection of TGFβ-MDSC could generate a long-lasting tumor immune response with potential benefit to survival [58,59].

The classification proposed by Keck et al. defined five subtypes of HNSCCs regardless of HPV status and named the phenotype of HNSCC rich in CD8+ “inflamed/mesenchymal”. Among HPV+ cancers, 63% are part of the “active immune class”. M1 tumor-associated macrophages (TAMs), antigen-presenting cells (APCs), and the large number of effector cells could be considered defining elements of this immune-related tumor subtype. The “exhausted tumor subtype” is associated with TGFβ and Wnt signaling (involved in stem cells and differentiation ability with consequences in the unfavorable prognosis). The “exhausted” percentage is considered much higher in HPV-HNSCC, with only 13% being in the HPV+ group. It is considered that the majority (67%) of HPV+ HNSCCs are associated with the immunologically active group, and only 5% are in the “exhausted” group [12,41,60,61,62].

## 5. Hypo-Fractionation—Currently in the Foreground

A regimen dedicated to older and frail patients proposed by the American Society for Radiation Oncology–European Society Radiation Oncology (ASTRO-ESTRO) consensus proposed hypo-fractionated radiotherapy (HFRT) as an alternative regimen to the standard protocol (68–70 Gy in 34–35 daily fractions). Two other regimens (60–66 Gy in 30 daily fractions and 55 Gy in 20 fractions, respectively) were compared with the standard regimen, and the results at 2 years were comparable between the three study groups. A dose per fraction of 2.4 Gy/fraction is the maximum at which the option of administering concurrent chemotherapy is considered possible. In the evaluation of doses, an equivalent dose in 2 Gy fractions (EQD2) and a biologically effective dose (BED) were evaluated for each regimen using *α*/*β* = 10Gy and *α*/*β* = 12Gy [63,64].

Huang et al. recommended to implement as standard a hypo-fractionated regimen with 2.4Gy/fraction for HPV+ T1-T3N0-N2c according to TNM-7 staging HNSCCs, HPV–T1-T2N0 HNSCCs, and also for selected cases of stage III HNSCCs in a pandemic context, which requires reducing the time spent by patients in the radiotherapy department [65].

Hypo-fractionated irradiation by the IMRT technique in total doses ≥ 50 Gy in 20 fractions for HNC was evaluated on a group of 56 patients. The results highlighted the absence of grade 4 and 5 acute and late toxicities and 79% and 25% rates of grade 2 and 3 acute and late toxicity, respectively. With a median OS of 46 months, the loco-regional control rate at 2 years was 87%. It should also be mentioned that the patients did not require a feeding tube or tracheostomy. Definitive or postoperative hypo-fractionated radiotherapy is considered to be well tolerated and without risks of unacceptable toxicity [66]. Re-irradiation in association with anti-PD-1 immunotherapy is considered an option with benefit in 25% of patients, with activation of the immunogenic cell death mechanism potentiated by hypo-fractionated radiotherapy. Koukourakis et al. evaluated the benefit and tolerance of an ultra-hypofractionated irradiation regimen in 17 cases of HNSCC, of which 4 cases had oligometastatic disease. Radiotherapy was delivered in 1, 2, and 3 fractions of 8Gy, and immunotherapy with nivolumab was delivered concurrently with radiotherapy and continued until the progression of the disease, the appearance of some immune-related adverse events, or up to a maximum number of 24 cycles. The average response rate was 70.6%, with a maximum after the regimen of two fractions. After 12 cycles of immunotherapy, 41.2% of patients had a complete response. The PFS and OS rates at 3 years were 35% and 50%, respectively. The authors consider this regimen a potential option to be evaluated in future studies [67].

The role of HPV status in cell death and in the expression of immune checkpoints was evaluated in four HNSCC cell lines using two irradiation regimens: a 5 × 3.0 Gy hypo-fractionated irradiation protocol and a 1 × 19.3 Gy single-dose irradiation protocol quantified with flow cytometry and polymerase chain reaction (PCR) shortly after irradiation. The hypo-fractionated regimen favored apoptosis in all cell lines regardless of HPV status, but necrosis was identified only in HPV-positive cell lines. In general, irradiation influenced immune-stimulatory checkpoints less than immunosuppressive ones, including PD-1 and PD-2, with the hypo-fractionation sequence generally favoring the surface expression of immune checkpoints regardless of HPV status. However, in the case of ICOS-L, the 5 × 3.0 Gy regimen induced a higher expression for HPV-positive cells. The authors conclude that HPV expression cannot be used as a single biomarker in anticipating a possible response to immunotherapy, but the expression of checkpoints after irradiation could be used for precision immunotherapy [47]. The failure of immunotherapy–radiotherapy combinations in HNSCC justified the initiation of preclinical studies to identify the causes and methods to combat this resistance. Targeting myeloid suppressors stimulated by irradiation does not demonstrate a benefit, but Treg depletion with anti-CD137 agonism and the combination of irradiation potentiates the antitumor immune response on HPV-negative HNSCC cell lines. The use of a hypo-fractionated regimen of 8 Gy × 5 irradiations in association with anti-CD25+ anti-CD137 demonstrated the activation of dendritic cell activation mechanisms in draining lymph nodes and a CD8+ T-lymphocyte-dependent response in the same study [68].

## 6. The Proposed LQ Model Adaptation

Considering these data, we propose an adaptation of the LQ model in which we add a term “equivalent to an additional dose” that can be lost or added as biological efficacy depending on the modulation induced on the immune response by irradiation (Equation (2)).

(2) $$BED=nd\left[1+\frac{d}{\frac{\alpha }{\beta }}\right]+Di$$

*Di* = t*^i^* × sb*^i^* × r × *i*;
t*^i^*—a term dependent on the moment of initiation of immunotherapy. In principle, this term could have higher values for the time interval of 24 h–10 days after the completion of radiotherapy;
sb*^i^*—a term dependent on the tumor’s immune phenotype;
r—the proposed fractionation regime;
*i*—a term dependent on the type of immunotherapy.

All the terms proposed could also have negative values that can cause a loss of the effectiveness of the synergistic treatment depending on the tilt of this scale in favor of immunosuppression or the antitumor immune effect. It should also be mentioned that, if more than one term has negative values, the general effect will be an amplified detrimental one and the second negative term must be included in the absolute size.

## 7. Conclusions

The new data regarding the involvement of the sixth “R” of radiobiology in HNSCC, especially for the HPV-driven subtype, but also for the “immune active” minority of HPV-negative HNSCC, bring to the fore a multifactorial variation of the *α*/*β* ratio. The involvement of the antitumor immune response and the dose/fractionation/volume factors as well as the therapeutic sequence in the case of multimodal treatments must be included as an additional term in the quadratic linear formalism for the adaptation of the LQ model in altered fractionation regimens and especially in hypo-fractionation settings.
